# Local Preirradiation of Infarcted Cardiac Tissue Substantially Enhances Cell Engraftment

**DOI:** 10.3390/ijms22179126

**Published:** 2021-08-24

**Authors:** Gloria Abizanda, Leyre López-Muneta, Javier Linares, Luis I. Ramos, Arantxa Baraibar-Churio, Miriam Bobadilla, Elena Iglesias, Giulia Coppiello, Purificación Ripalda-Cemboráin, Xabier L. Aranguren, Felipe Prósper, Ana Pérez-Ruiz, Xonia Carvajal-Vergara

**Affiliations:** 1 Regenerative Medicine Program, Foundation for Applied Medical Research (FIMA), University of Navarra, Instituto de Investigación Sanitaria de Navarra (IdiSNA), 31008 Pamplona, Spain; gabizanda@unav.es (G.A.); llopez.13@alumni.unav.es (L.L.-M.); jlinares@alumni.unav.es (J.L.); abaraibarc@alumni.unav.es (A.B.-C.); mbobadillam@alumni.unav.es (M.B.); eiglesias@unav.es (E.I.); gcoppiello@unav.es (G.C.); pripalda@unav.es (P.R.-C.); xlaranguren@unav.es (X.L.A.); fprosper@unav.es (F.P.); 2 Department of Oncology, Clínica Universidad de Navarra, 31008 Pamplona, Spain; liramos@unav.es; 3 Centro de Investigación Biomédica en Red de Cáncer (CIBERONC), 28029 Madrid, Spain; 4 Department of Hematology and Cell Therapy, Clínica Universidad de Navarra, 31008 Pamplona, Spain

**Keywords:** myocardial infarction, cell therapy, local irradiation, cell engraftment

## Abstract

The success of cell therapy for the treatment of myocardial infarction depends on finding novel approaches that can substantially implement the engraftment of the transplanted cells. In order to enhance cell engraftment, most studies have focused on the pretreatment of transplantable cells. Here we have considered an alternative approach that involves the preconditioning of infarcted heart tissue to reduce endogenous cell activity and thus provide an advantage to our exogenous cells. This treatment is routinely used in other tissues such as bone marrow and skeletal muscle to improve cell engraftment, but it has never been taken in cardiac tissue. To avoid long-term cardiotoxicity induced by full heart irradiation we developed a rat model of a catheter-based heart irradiation system to locally impact a delimited region of the infarcted cardiac tissue. As proof of concept, we transferred ZsGreen^+^ iPSCs in the infarcted heart, due to their ease of use and detection. We found a very significant increase in cell engraftment in preirradiated rats. In this study, we demonstrate for the first time that preconditioning the infarcted cardiac tissue with local irradiation can substantially enhance cell engraftment.

## 1. Introduction

Cardiovascular diseases are the leading cause of mortality and morbidity worldwide [1,2]. Acute myocardial infarction (AMI) occurs when coronary blood flow is decreased or obstructed. Cardiac ischemia causes the loss of millions of cardiac cells which is compensated through hypertrophy and fibrosis leading to a non-functional scar [3]. Ultimately, the heart is not able to pump sufficient blood to meet the body’s needs, and heart failure ensues [4]. Pharmacological therapy has decreased heart failure-associated mortality by limiting the adverse remodeling process; however, many severe cases still require heart transplantation which is limited by a shortage of donors. In this scenario, cell therapy emerged two decades ago as a promising treatment to regenerate and repair the damaged heart. Since then, multiple cell types have been assayed pre-clinically and clinically, mostly bone marrow stem cells, and more recently cells with greater cardiomyogenic potential such as pluripotent stem cell-derived cells [5,6,7]. Unfortunately, cell therapy has shown only modest long-term functional benefit [5,6,7]. The greatest and most widespread problem is that the cells delivered are poorly retained in the transplanted area, with most remaining cells dying within hours or days of transplantation [8,9]. Furthermore, the few surviving cells mainly have a paracrine effect. Thus, novel approaches or technologies aimed at improving donor cell survival, engraftment and cardiac tissue *de novo* regeneration are urgently needed. Recent research efforts have focused on preconditioning transplantable cells to overcome this stressful environment where donor cells face the inflammatory and fibrotic tissue with low available concentrations of oxygen and glucose that complicate their survival [10,11]. However, it is still early to have a clear idea of the true extent and impact of these new methods.

Alternatively, preconditioning the infarcted heart before cell transfer would also improve retention and function of therapeutic cells. In line with this, pre-clinical studies in mouse models demonstrate that local irradiation of a range of tissues such as bone marrow, skin or skeletal muscle prior to cell transplantation potentiates cell engraftment [12,13,14]. However, there is no evidence of the use of local irradiation of cardiac tissue for this purpose. Radiotherapy is routinely used to effectively treat different types of tumors based on the DNA damage caused by ionizing irradiation in proliferating cells. Furthermore, following new advances in radiotherapy, currently, it is possible to locally treat the affected area more precisely. Brachytherapy is a very site-specific procedure in which the source of radiation is placed inside the body to treat the target area as closely as possible, thus avoiding any impact on neighboring tissues and organs [15].

We propose the use of brachytherapy to locally impact a delimited region of the infarcted cardiac tissue, that will become fibrotic and non-functional. In this study, we have developed a rat model of a catheter-based heart irradiation system and demonstrate that local irradiation of infarcted cardiac tissue can increase cell engraftment.

## 2. Results

**Figure 1:**
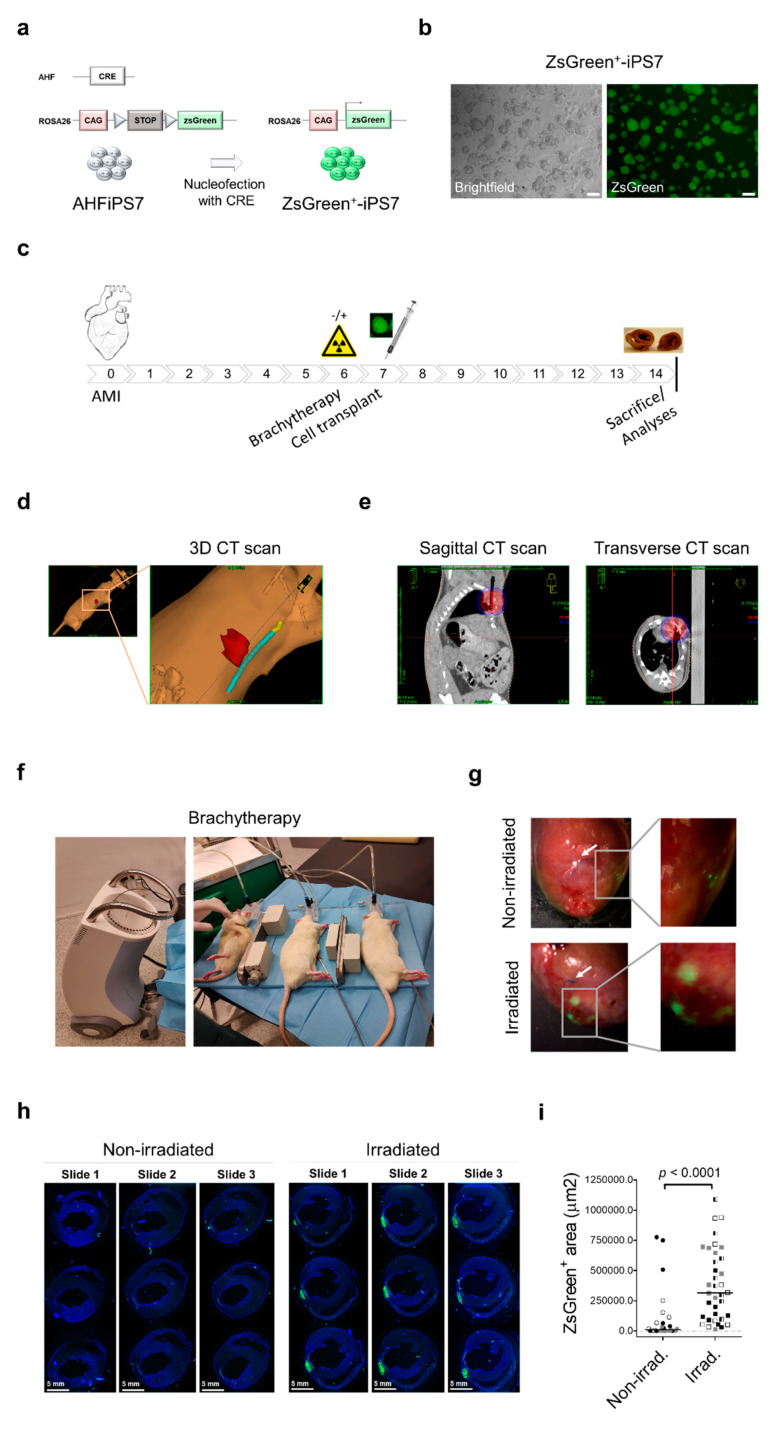
Local irradiation of infarcted cardiac tissue potentiates ZsGreen^+^-iPS7 cell engraftment. (**a**) Graphical overview: ZsGreen^+^-iPS7 were derived from AHFiPS7 cells by nucleofection of pBS185 Cre plasmid. Scale bars, 100 µm. (**b**) Expression of ZsGreen in ZsGreen^+^-iPS7 cells under a fluorescence microscope. Scale bars, 100 µm. (**c**) Graphical overview of the experimental procedure. (**d**) Three-dimensional reconstruction of CT images in a rat in which the region to be treated is represented in red and the catheter in blue (upper panels). Dots inside the catheter represent positions where the source can be placed. (**e**) Sagittal and transverse views of the CT scan in which dose distribution is depicted by red and blue areas, receiving 20 and 10 Gy, respectively (bottom panels). (**f**) Picture of three rats connected to the afterloader system (Elekta Flexitron) using the implanted catheter through which the Ir-192 radioactive source is inserted into the animal. (**g**) ZsGreen expression in a heart from the non-irradiated and irradiated group under fluorescence magnifying lamp upon exposure to blue light. White arrows point to the suture threads that were used for the permanent ligation of the descending coronary artery to induce AMI. (**h**) ZsGreen and DAPI fluorescence signals were scanned using an automated quantitative pathology imaging system and representative sections of a heart from the non-irradiated and irradiated group are shown. Scale bars, 5 mm. (**i**) Quantification of ZsGreen^+^ areas from both the non-irradiated (*n* = 3) and irradiated (*n* = 4) groups and the medians of each data group are represented (Shapiro-Wilk normality test *p* < 0.05). Non-irradiated group vs. irradiated group *p* < 0.0001 using two-tailed, Mann-Whitney U test.

We used AHFiPS7, previously established and described by our group [16], as donor transplantable cells, where CRE recombinase was nucleofected to express ZsGreen under control of ubiquitous CAG promoter specifically inserted at the ROSA26 locus (Figure 1a,b and Supplementary Materials Figure S1). Thus, we were able to readily obtain large quantities of ZsGreen positive (ZsGreen^+^) donor cells and detect them in the recipient hearts without immunostaining (Supplementary Materials Figure S2), which greatly facilitated the quantification of engrafted cells.

The experimental procedure carried out in this study is depicted in Figure 1c. Specifically, AMI was induced in adult Sprague-Dawley rats by permanent ligation of the descending coronary artery as described previously [17]. In the same surgical procedure, a catheter was placed adjacent to the left ventricle and fixed between the 3rd and 5th intercostal spaces. The correct positioning of the catheter was verified by computed tomography and the treatment plan was designed (Figure 1d,e and Supplementary Materials Figure S3).

One week after AMI, one million ZsGreen^+^-iPS7 cells were intramyocardially transplanted. The day before cell transplantation, four rats underwent high dose-rate brachytherapy (Figure 1f), receiving local irradiation in a delimited region proximal to the catheter (area and dose depicted in Figure 1e and Supplementary Figure S3b). Four non-irradiated rats were used as controls. All the animals were treated with immunosuppressants (tacrolimus and anti-asialo-GM1) to prevent cell rejection mediated by T-lymphocytes and natural killer cell activity. One control recipient (Rat#4) did not survive until the end of the experiment, probably due to complications associated with AMI or immunosuppression. The remaining recipients were sacrificed one week after cell transplantation and ZsGreen expressing areas were visualized under a fluorescent magnifying lamp in the isolated hearts with these areas being more apparent and brighter in the irradiated recipient-derived hearts (Figure 1g). Then, the hearts were cut just above the suture thread, which marked the coronary ligation, and serially cryo-sectioned for histological analyses.

Major complications or adverse events caused by local irradiation were not observed and cardiac tissue microstructure was preserved in irradiated rats at the time of cell engraftment analyses (Supplementary Materials Figure S4). All sections were analyzed under a fluorescence microscope to select those slides containing sections with ZsGreen^+^ area/s. Three consecutive slides per rat containing 9 heart sections, representative of 1.67 mm of heart length, were scanned using an automated quantitative pathology imaging system, where the central slide included the largest number of sections with ZsGreen^+^ area/s (Supplementary Materials Figure S5 and Figure S6). Representative heart sections from an irradiated and non-irradiated rat are shown in Figure 1h. Quantification of total ZsGreen^+^ areas demonstrated that ZsGreen^+^-iPS7 engrafted cells were significantly more abundant in the irradiated group compared to the control recipients (*p* < 0.0001; median.irradiated group = 315,263 µm^2^ vs. median.control group = 12,093 µm^2^), (Figure 1i). These data demonstrate that local preirradiation of infarcted cardiac tissue significantly increases cell engraftment.

## 3. Discussion

By using local preirradiation, we achieved a 26-fold greater engraftment rate than in the non-irradiated group. It is known that irradiation can enhance cell engraftment in other tissues, by the incapacitation of endogenous cells that compete with exogenous cells for the niche occupancy [12], in a dose- and frequency-dependent manner [12,14]. On the other hand, AMI causes a continuous cardiac remodeling process and different cell populations, including inflammatory cells or cardiac fibroblasts at different activation states, are present or enriched on a given day after injury [18]. Thus, it is reasonable to think that precise local irradiation at the site of the infarcted heart tissue may lead deleterious dividing cells to accumulate DNA damage and die, thus facilitating donor cell engraftment. If this is the situation, it would be interesting to explore in future studies whether irradiation, beyond improving cell engraftment, may also impair the process of cardiac remodeling. We are fully aware that these cells do not represent a good cell candidate to improve heart function. However, the sole objective of this study was to demonstrate that pre-treatment of infarcted cardiac tissue by local irradiation can increase cell engraftment, as others previously proved the validity of this approach in skeletal muscle using different tumorigenic cells [19]. This is the first demonstration that local preirradiation can significantly enhance cell engraftment in cardiac tissue and offers a great opportunity to test different types of cells and improve the effectiveness of cell therapy in the cardiovascular field.

## 4. Materials and Methods

### 4.1. ZsGreen-iPS7 Cells Derivation and Culture Conditions

Mouse iPSCs were cultured in DMEM, 15% KnockOut serum replacement, 0.1 mM 2-mercaptoethanol, 2 mM GlutaMAX, 0.1 mM MEM-NEAA, 100 U/mL Pen–100 µg/mL Strep (all from Gibco), and 103 U/mL LIF (Millipore) and seeded onto irradiated mouse embryonic fibroblasts (ɣMEFs) on 0.1% gelatin-precoated plates. The media was changed every other day.

AHFiPS7, previously established and described by our group [16], were nucleofected with 6 µg of pBS185 CRE plasmid (Addgene #11916) using the Amaxa nucleofection kit (VPH-5012) with one pulse of program A13 of Amaxa Nucleofector II to obtain ZsGreen positive iPS7 (ZsGreen^+^-iPS7) cells. ZsGreen^+^ cells were sorted by FACSAria (Figure S1).

### 4.2. Experimental Animals

A total of 10 male Sprague-Dawley 11-week-old rats (Janvier Labs) were used in this study. All rats underwent permanent occlusion of the left anterior descending coronary artery, as previously described [17]. Briefly, rats were anesthetized with Isoflurane (IsoVet-B. Braun), intubated and mechanically ventilated. Prior to surgery, animals received analgesic therapy with Fentanyl (FENTANEST, Kern Pharma, S.L. 300 µg/kg intraperitoneally) and 1% lidocaine/0.25% bupivacaine (B. Braun, both 1 mg/kg subcutaneously) infiltrated the skin and underlying connective tissue planes. The heart was accessed through a left thoracotomy through the 4th intercostal space, and the left anterior descending coronary artery was permanently occluded 2–3 mm distal to its origin. After stabilization of the heart, the irradiation catheter was put in place. The distal end was fixed in the 3rd intercostal space with 7/0 Prolene (Ethicon, Bridgewater, NJ, USA) and the proximal end was exteriorized through the 5th intercostal space and was tunneled subcutaneously to the lower abdomen, where it was fixed with the same suture material. After checking the stability of the animal, the chest was then closed in layers, and the rats were allowed to recover on a heating pad.

Eight out of ten rats survived the infarct surgery. One week after acute myocardial infarction, rats underwent the same procedure with the same left lateral thoracotomy and under the same anesthetic conditions. Rats were allocated to receive one million ZsGreen^+^-iPS7 cells per animal. The cells were suspended in 9 µL of PBS, loaded in a Hamilton syringe (701 N, 10 µL) and were delivered into the infarcted tissue by three proximal injections of 3 µL each, two in the peri-infarct zone and one in the infarcted area. The brachytherapy catheter was previously removed.

To prevent immune rejection, since ZsGreen^+^-iPS7 cells are of mouse origin, rats were immunosuppressed by the administration of tacrolimus (Prograf, 1 mg/kg per day) every 24 h until sacrifice, starting two days before cell transplantation, to suppress T lymphocyte activity and anti-asialo-GM1 antiserum (Wako, following the manufacturer´s recommendation) before cell transplantation and 4 days later to prevent natural killer cell activity.

On the day of sacrifice, animals were anesthetized by intraperitoneal injection of ketamine (Imalgene, 80 mg/kg) and dexmedetomidine (Orion Pharma (Espo, Finland), 150 µg/kg). Heparin (10 U/100 g) was used to prevent excessive coagulation during the thoracotomy. Transcardial perfusion was performed with 50 mL of ice-cold PBS solution and 50 mL of cold 4% paraformaldehyde (PFA) to wash the vascular system and fix the heart using a flow pump set at a 5 mL/minute rate. Immediately after perfusion, the heart was excised, analyzed under a fluorescence magnifying lamp, and further processed as described below for histological analyses.

### 4.3. Tissue Processing and Staining

Hearts were cut right above the suture thread using an acrylic rat heart slicer matrix (Zivic Instruments, Pittsburgh, PA, USA) and both portions were fixed in 4% PFA for 4 h, washed three times in PBS for 15 min, and immersed into 15% sucrose/PBS for 30 min and then into 30% of sucrose/PBS overnight at 4 °C. Finally, heart fragments were embedded in O.C.T. compound (Tissue-Tek), frozen on dry ice and stored at −80 °C.

For histological analysis, hearts were serially cryosectioned in 10-μm transversal sections that were collected within each 200 µm, as indicated in Figure S4a. Haematoxylin and eosin (H&E) histological staining was performed by Morphology Core Facility at CIMA. Detection of tissues containing cells of donor origin was based on the presence of ZsGreen^+^ signals under an epifluorescence microscope (Zeiss Axiophot, Jena, Germany) with no need for immunostaining. Selected slides were mounted in Faramount fluorescent mounting medium containing 4,6-diamidino-2-phenylindole (DAPI; 100 ng/mL; Molecular Probes, Eugene, OR, USA) to detect all nuclei.

ZsGreen fluorescence signal detection and appropriate tissue preservation were assessed previously using mouse hearts (Figure S2). Briefly, ZsGreen^+^-iPS7 cells were implanted into healthy mouse hearts and immediately perfused and processed for histological analyses as described above. Tissue cryosections were air-dried and then blocked in PBS containing 10% goat serum, 1.5% BSA and 0.5% Triton X-100 (blocking solution) for 1 h at room temperature. Immunolabeling was performed overnight at 4 °C with antibodies against α-smooth muscle actin (α-SMA) [rabbit polyclonal antibody, Abcam ab5694] and cardiac troponin T (cTnT) (mouse monoclonal antibody, Thermo Fisher Scientific MS-295-P0, Waltham, MA, USA) diluted in blocking solution (1:200 and 1:100, respectively). Then, tissue sections were washed in PBST (PBS containing 0.05% Tween 20) and incubated with goat anti-rabbit AlexaFluor-647 (Invitrogen, A21244, Waltham, MA, USA) and goat anti-mouse AlexaFluor-546 (Invitrogen, A11030) secondary antibodies, diluted 1:1000 in PBS. All tissue sections were washed before being mounted in DAPI and analyzed under an automated fluorescence microscope (Zeiss Axio Imager M1).

### 4.4. Brachytherapy in Rats

Rats were irradiated using brachytherapy. The radioactive source (Ir-192) was connected to an afterloader system (Elekta Flexitron), which places the source in the right position inside the rat for a short time. The correct positioning of the catheter implanted in the rats with regard to the target region was verified using a computed tomography (CT) scan (Siemens Sensation 16).

The scanning protocol is shown in Table 1:

The CT images (Figure S3) were used to design a treatment plan to give the desired radiation dose to the target zone, minimizing dose application to non-target sites, by using specically designed software (Oncentra v4.5.2). This program calculates the stop positions and the time spent with the source to reach the required dose radiation distribution in the target zone and the rest of the body. The plan was designed to give at least 8 Gy to the target volume delimited in the CT. Once the treatment design was finished, all the necessary data were digitally transferred to the afterloader system. On the day of irradiation, the catheter implanted in the rat was connected to this system and the treatment was begun.

### 4.5. Fluorescence Microscopy and ZsGreen Quantification

All slides were analyzed under fluorescence microscopy (Zeiss Axiophot epifluorescence microscope) and those containing ZsGreen^+^ areas were selected. For quantitative analyses, we selected the slide containing the section with the largest number of ZsGreen^+^ area/s, and the following previous and subsequent slides. Thus, we analyzed 9 heart sections (3 sections/slide) in each recipient rat, which represents 1.67 mm of the length of the heart (Figure S4a). ZsGreen and DAPI fluorescence signals were scanned in an automated quantitative pathology imaging system (Vectra Polaris, Perkin Elmer) at the same exposure time. Images were edited and quantification of ZsGreen^+^ area/s in each tissue section was carried out by a researcher blind to sample origin by using the Fiji-Image J software.

### 4.6. Statistical Analysis

ZsGreen^+^ areas from both groups, non-irradiated (26 values, 3 rats) and irradiated (36 values, 4 rats), together with the median of each data group were calculated. Statistical analyses were performed using GraphPad Prism software. The median of each data group is shown since data did not show a normal distribution (Shapiro-Wilk normality test *p* < 0.05). Statistical comparison between the two groups was performed using two-tailed, Mann-Whitney U tests. The p-value (*p* < 0.0001) obtained was considered to indicate a very significant difference between the two groups.

## Figures and Tables

**Table 1:** The scanning conditions used are detailed.

Parameters of the Scanning Protocol	
Mode	Axial
Pitch	0
KVp	80
mA	320
Rotation time (s)	1
mAs	320
FOV (cm)	20
Reconstruction diameter (cm)	20
Slice thickness (mm)	1
Convolution kernel	H31s

## Supplementary Materials

The following are available online at https://www.mdpi.com/article/10.3390/ijms22179126/s1.
