# Supplementary material for: Local Preirradiation of Infarcted Cardiac Tissue Substantially Enhances Cell Engraftment
Source: Int J Mol Sci. 2021 Aug 24;22(17):9126. doi: 10.3390/ijms22179126 (PMC8430717; doi:10.3390/ijms22179126)
Supplement: Supplementary file 1 [file ijms-22-09126-s001.zip › ijms-1315419-supplementary.pdf]

International Journal of *Molecular Sciences*

MDPI

Supplementary materials for:

Short Communication

Local Preirradiation of Infarcted Cardiac Tissue Substantially Enhances Cell Engraftment

Gloria Abizanda [1,†], Leyre López-Muneta [1,†], Javier Linares [1,†], Luis I. Ramos [2], Arantxa Baraibar-Churio [1], Miriam Bobadilla [1], Elena Iglesias [1], Giulia Coppiello [1], Purificación Ripalda-Cemboráin [1], Xabier L. Aranguren [1], Felipe Prósper [1,3,4], Ana Pérez-Ruiz [1,*] and Xonia Carvajal-Vergara [1,*]

[1] Regenerative Medicine Program, Foundation for Applied Medical Research (FIMA), University of Navarra, Instituto de Investigación Sanitaria de Navarra (IdiSNA), 31008 Pamplona, Spain; gabizanda@unav.es (G.A.); llopez.13@alumni.unav.es (L.L.-M.); jlinares@alumni.unav.es (J.L.); abaraibarc@alumni.unav.es (A.B.-C.); mbobadillam@alumni.unav.es (M.B.); eiglesias@unav.es (E.I.); gcoppiello@unav.es (G.C.); pripalda@unav.es (P.R.-C.); xlaranguren@unav.es (X.L.A.); fprosper@unav.es (F.P.)

[2] Department of Oncology, Clínica Universidad de Navarra, 31008 Pamplona, Spain; liramos@unav.es

[3] Centro de Investigación Biomédica en Red de Cáncer (CIBERONC), 28029 Madrid, Spain

[4] Department of Hematology and Cell Therapy, Clínica Universidad de Navarra, 31008 Pamplona, Spain

* Correspondence: aperu@unav.es (A.P.-R.); xcarvajal@unav.es (X.C.-V.); Tel.: +34 948194700

† Equally contributing authors.

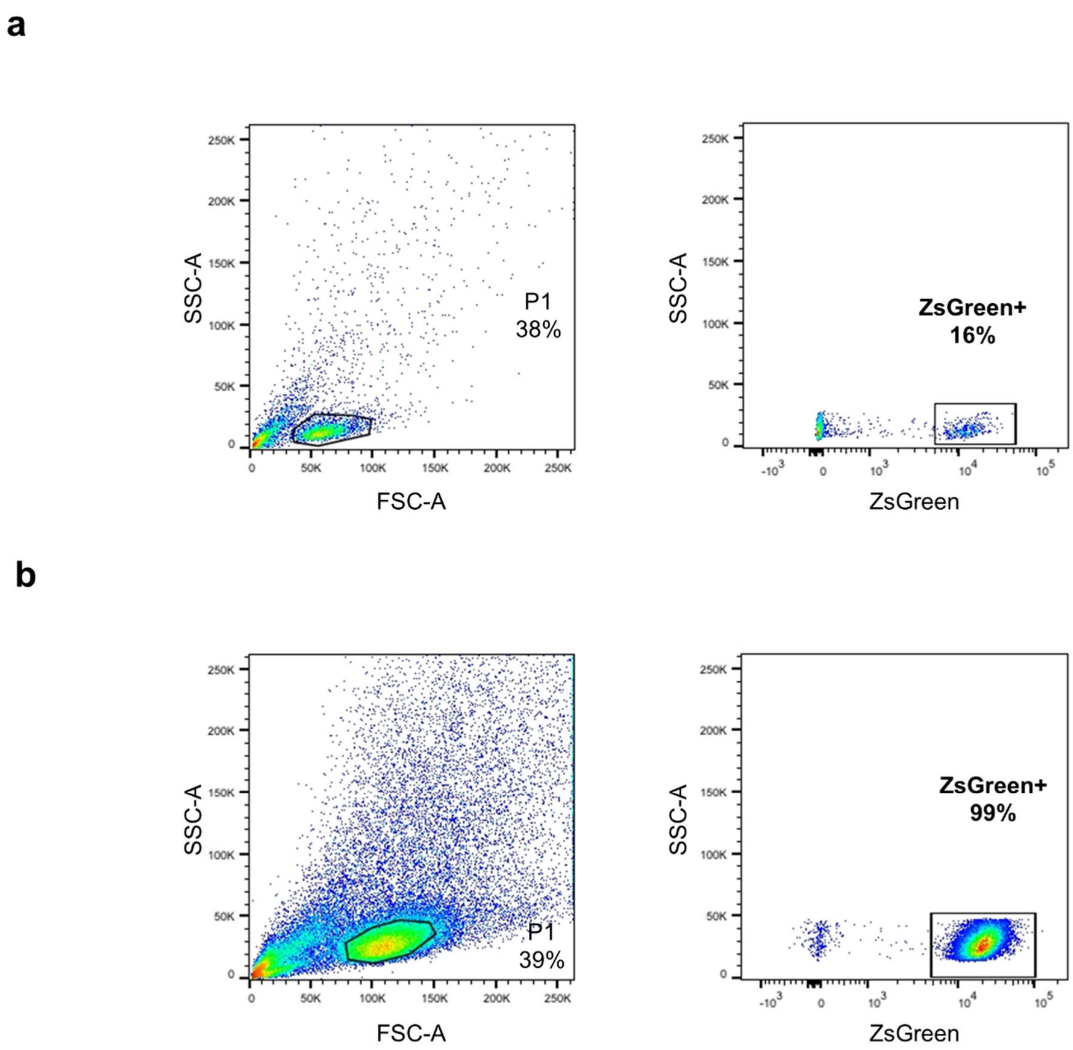

Supplementary materials, Figure S1. ZsGreen+-iPS7 fluorescence-activated cell sorting (FACS) analyses. AHFiPS7 cells were nucleofected with pBS185 CRE plasmid and then subjected to cell sorting to obtain a pure ZsGreen+-iPS7 cell line. Dot-plots corresponding to the pre-sorting ZsGreen+ cells selection **(a)** and post sorting analysis **(b)** are shown.

a

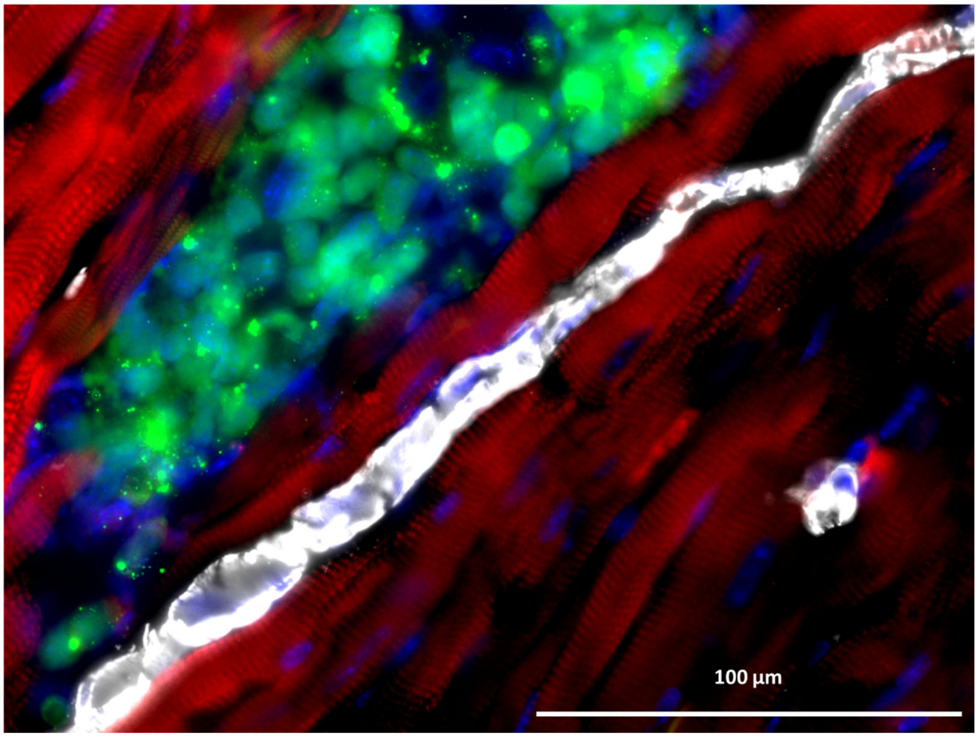

Supplementary materials, Figure S2. Immunohistochemical analysis of cardiac tissue after ZsGreen+-iPS7 cell injection. **(a)** A Hamilton syringe was used to intramyocardially inject ZsGreen+-iPS7 cells into a healthy mouse heart which was immediately perfused and processed for histological analyses. ZsGreen expression was analyzed under a fluorescence microscope without immunostaining. Tissue preservation was verified by immunostaining for anti-SMA (in white) and anti-cTnT (in red) that marked blood vessels and cardiomyocytes, respectively. Cell nuclei (in blue) as detected by DAPI.

a

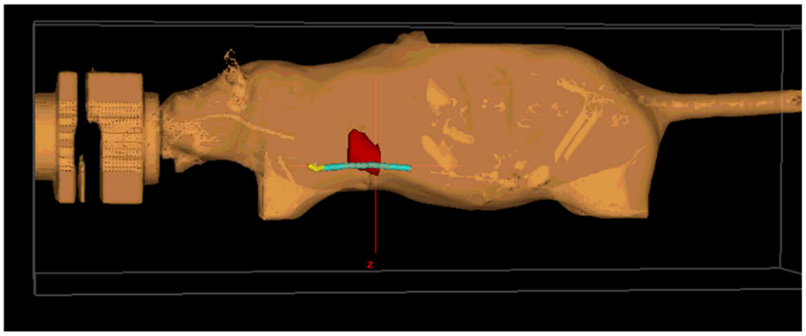

b

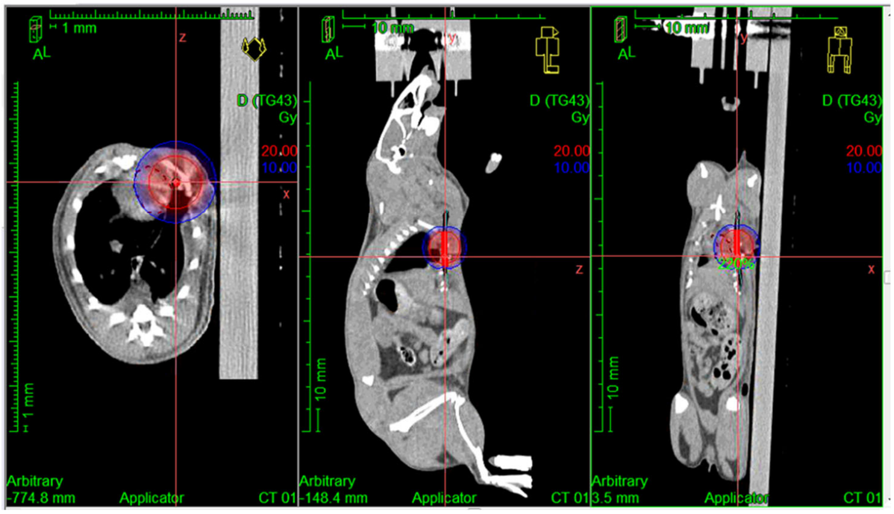

Supplementary materials, Figure S3. Computed tomography (CT) analysis to design the treatment plan in rats. **(a)** Three-dimensional reconstruction of a rat using CT images. The catheter is marked in blue and the beige dots inside represent the sites where the irradiation source can be placed to treat the target region, represented in red. **(b)** Transverse, sagittal and frontal view of the CT scan. Areas in red and blue delimit the targeted regions receiving 20 and 10 Gy, respectively. Only the positions marked as red dots were used to deliver the irradiation.

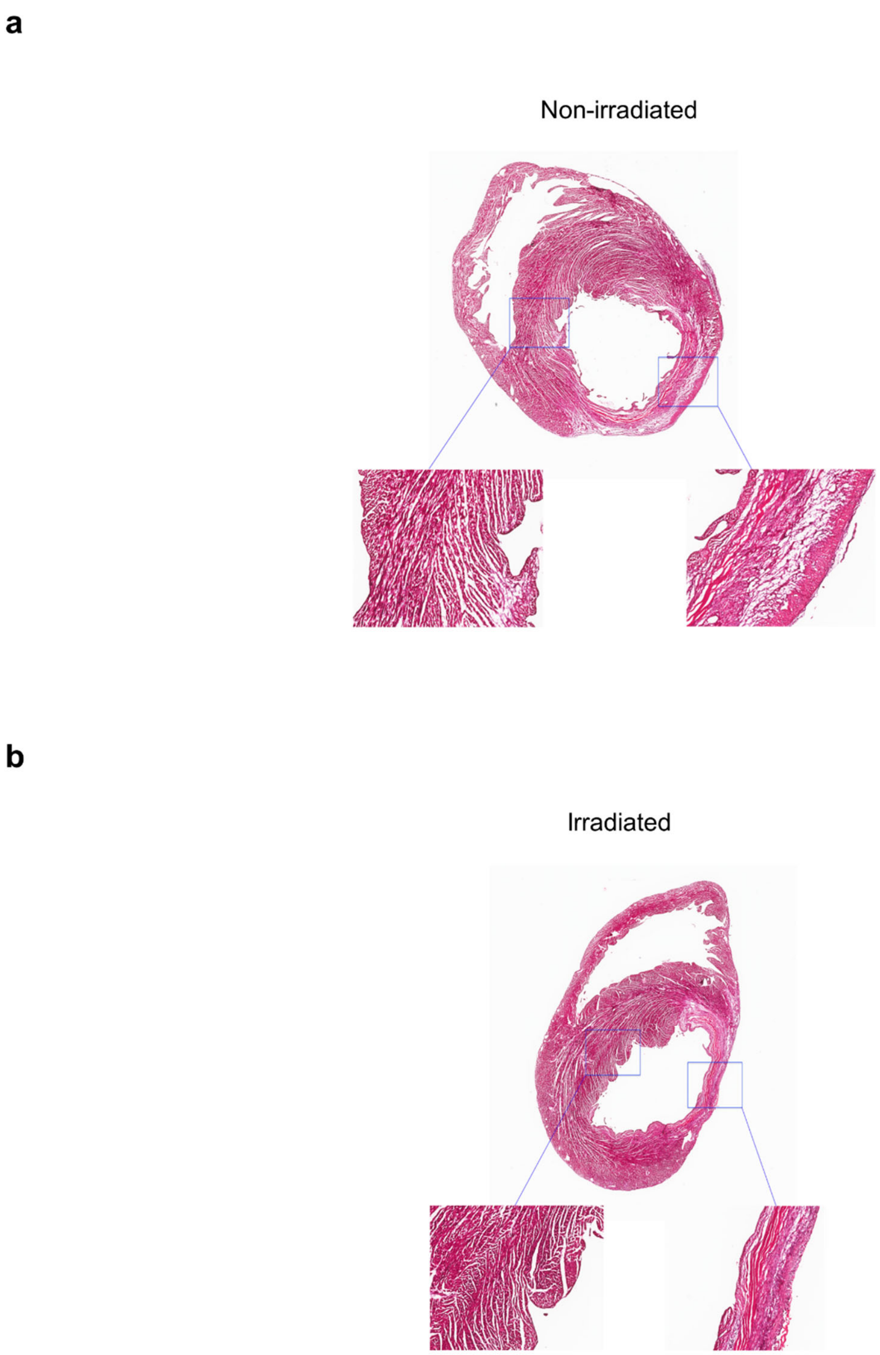

Supplementary materials, Figure S4. Cardiac tissue microstructure at the time of cell engraftment anlyses. H&E staining of representative histological sections of a heart from the non-irradiated **(a)** and irradiated **(b)** group is shown.

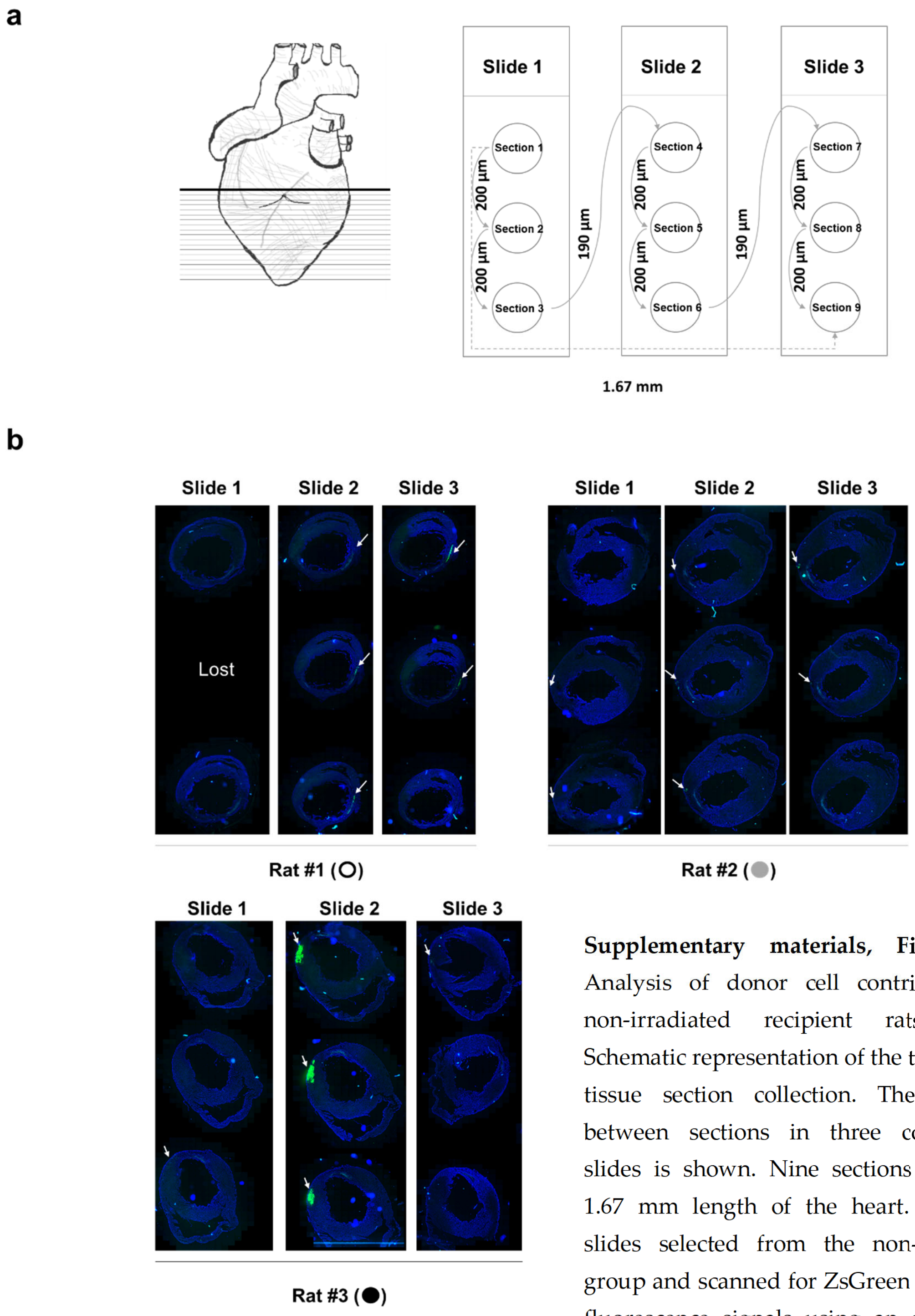

Supplementary materials, Figure S5. Analysis of donor cell contribution in non-irradiated recipient rats. **(a)** Schematic representation of the transversal tissue section collection. The distance between sections in three consecutive slides is shown. Nine sections represent 1.67 mm length of the heart. **(b)** The slides selected from the non-irradiated group and scanned for ZsGreen and DAPI fluorescence signals using an automated quantitative pathology imaging system are shown. White arrows point to ZsGreen^{+} areas.

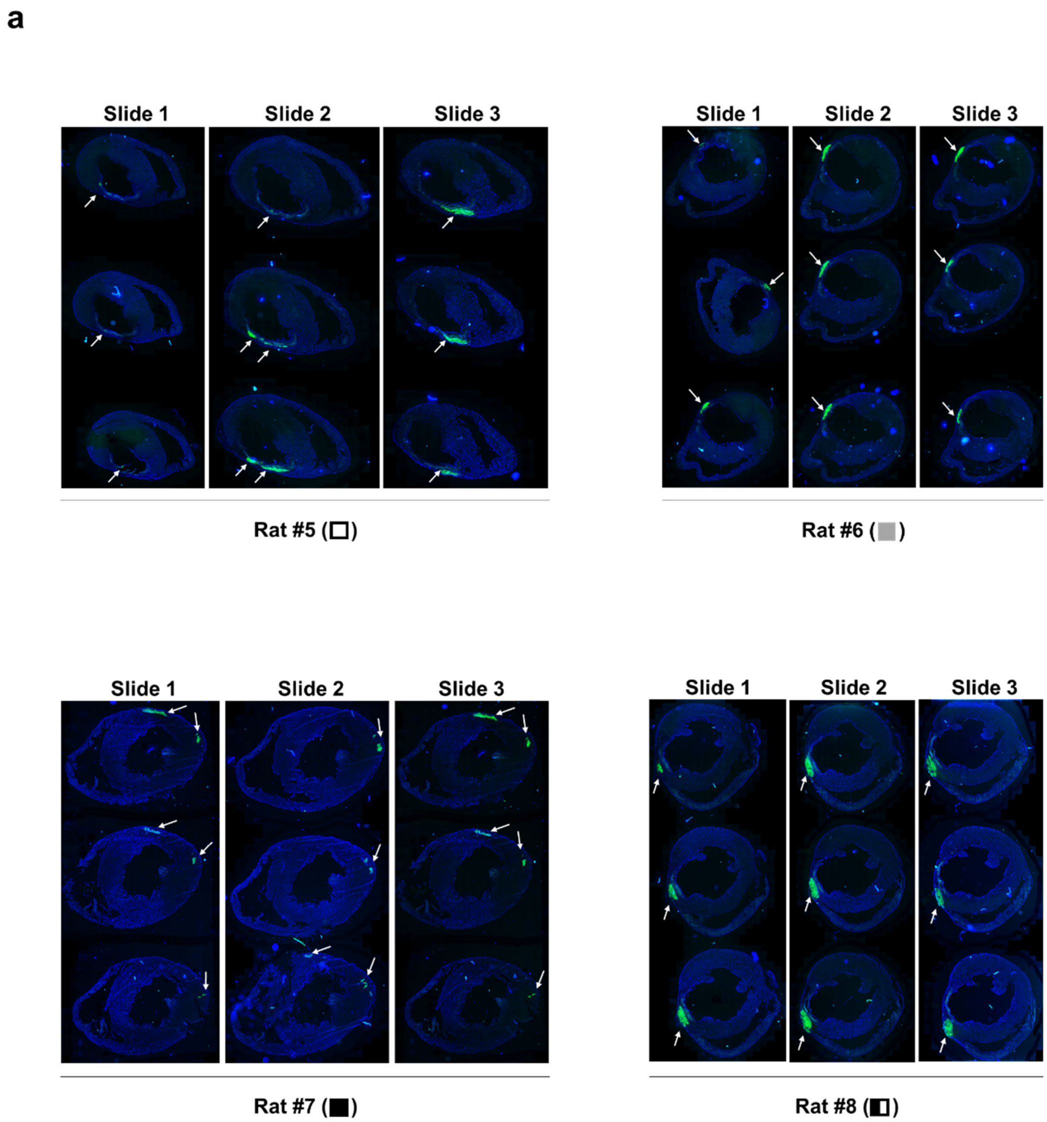

Supplementary materials, Figure S6. Analysis of donor cell contribution in irradiated recipient rats. **(a)** Images show ZsGreen and DAPI expression in the selected slides from the irradiated group. White arrows point to ZsGreen+ areas, present in all sections.
